# Limitations of two time point data for understanding individual differences in longitudinal modeling — What can difference reveal about change?

**DOI:** 10.1016/j.dcn.2024.101353

**Published:** 2024-02-05

**Authors:** Sam Parsons, Ethan M. McCormick

**Affiliations:** a Cognitive Neuroscience Department, Donders Institute for Brain, Cognition and Behavior, Radboud University Medical Center, Nijmegen, The Netherlands; b Methodology & Statistics Department, Institute of Psychology, Leiden University, Leiden, The Netherlands

**Keywords:** Developmental neuroscience, Longitudinal analyses, Trajectories of change, Simulation, Two timepoints, Individual differences

## Abstract

Emerging neuroimaging studies investigating changes in the brain aim to collect sufficient data points to examine trajectories of change across key developmental periods. Yet, current studies are often constrained by the number of time points available now. We demonstrate that these constraints should be taken seriously and that studies with two time points should focus on particular questions (e.g., group-level or intervention effects), while complex questions of individual differences and investigations into causes and consequences of those differences should be deferred until additional time points can be incorporated into models of change. We generated underlying longitudinal data and fit models with 2, 3, 4, and 5 time points across 1000 samples. While fixed effects could be recovered on average even with few time points, recovery of individual differences was particularly poor for the two time point model, correlating at *r* = 0.41 with the true individual parameters - meaning these scores share only 16.8% of variance As expected, models with more time points recovered the growth parameter more accurately; yet parameter recovery for the three time point model was still low, correlating around *r* = 0.57. We argue that preliminary analyses on early subsets of time points in longitudinal analyses should focus on these average or group-level effects and that individual difference questions should be addressed in samples that maximize the number of time points available. We conclude with recommendations for researchers using early time point models, including ideas for preregistration, careful interpretation of 2 time point results, and treating longitudinal analyses as dynamic, where early findings are updated as additional information becomes available.

## 1 Introduction

The prevalence of longitudinal data is increasing rapidly (Kievit et al., 2022), bringing a new lens to bear on understanding the origins, patterns across time, and consequences of individual differences in developmental trajectories. Specifically, studies with multiple waves of data collection across weeks, months, and years allow researchers to capture within-person change and investigate developmental processes (Bollen and Curran, 2006, Curran et al., 2010).^1^ However, the long-term nature of longitudinal data collection presents a dilemma for researchers faced with practical career-related concerns for themselves, grant stakeholders, and students/trainees — to publish early results on early waves of an ongoing study or to wait until data collection is complete. We take a pragmatic view that this is a **real tradeoff** for all researchers and that career-related concerns of graduation, promotion, and continued funding are pressing concerns that we are all subject to. On the other hand, we cannot ignore potential theoretical and statistical issues with modeling longitudinal processes on early waves of data. As such, we need to develop heuristics for which kinds of questions can be reasonably addressed in these early waves of data – for instance fixed effects questions or experimental manipulations – and which are more properly left for models which include additional repeated measurements. In particular, we focus here on investigating the limitations of analyzing two waves of data as a proxy for a full longitudinal trajectory model within the context of developmental cognitive neuroscience research and highlight alternative theoretical questions which these models may be better suited for.

Large multi-site longitudinal studies are a mainstay of developmental, health, and educational research (e.g., National Longitudinal Survey of Youth [ NLSY]; Add Health (Harris et al., 2019); National Head Start Impact Study). In recent years, there has been major research funding investment into longitudinal studies including neuroimaging and neurocognitive measurements. Exemplars of these emerging studies include the Adolescent Brain Cognitive Development (ABCD; Casey et al., 2018), Healthy Brain and Child Development Study (HCBD; Volkow et al., 2021), and UK Biobank (Sudlow et al., 2015; according to a recent announcement). While these studies may ultimately collect several waves of data, spanning larger ranges of the lifespan, researchers currently have access to early waves, and face the decision of whether to analyze and publish findings based on the available data. In determining which kinds of questions we can successfully ask using these data, we need to address several questions: (1) does the model we might fit with these early waves of data conform to our theoretical models of the process of change under consideration? and (2) are the kind of effects of interest (e.g., group versus individual differences) reliably captured by the model we are able to fit to early time points of data.

To assess the theoretical validity of using early time points as indicators of the true developmental effects across the full longitudinal process, one key indicator we can look to is the planned nature of the study itself, and the kinds of effects being estimated. In many clinical applications, pre-post, 2-wave longitudinal designs are very common, often with a between-group comparison of treatment and control, and the general reliability of change scores has attracted much attention historically (Rogosa and Willett, 1983, Willett, 1988). While questions have been raised about how well this design captures an underlying change process (Shadish et al., 2002), the design clearly articulates a theory of change that can be tested with standard methods (ANOVA, ANCOVA, etc.; Bonate, 2000). Another type of theoretical question that remains common relates to modeling the developmental trajectory of a given measure (e.g., reward processing, cognitive control) where the emphasis is on describing group-level trends over developmental time (e.g., Mills et al., 2016, Braams et al., 2015, McCormick et al., 2021, Quach et al., 2020), and where time lag influences how reliably two time points capture a change processes (McArdle and Woodcock, 1997, Brandmaier et al., 2015). Here the relationship between the number of available waves of data is more complex — although more time points are undoubtedly better, the developmental coverage of the available data is also a relevant factor. For instance, in early papers on the BrainTime study (e.g., Braams et al., 2015, van Duijvenvoorde et al., 2016, Peters et al., 2016) or in lifespan work (Sørensen et al., 2021), each participant had only two waves of data, but developmental coverage extended over decades of life, allowing for inferences at the group level about the average developmental trajectory (see McCormick et al., 2023 for relevant comparisons of accelerated vs. cohort longitudinal designs).

However, a major focus of modern developmental cognitive neuroscience is that of understanding individual differences in both trajectories of change over time, as well as relevant predictors or downstream consequences of these differences. It is here that the most caution is warranted with early waves of data since the reliability of these individual differences is directly impacted by the availability of data at the individual level to estimate these effects. If in practice, we cannot reliably capture the same information on individual differences in developmental trajectories we might get from the full (or at least fuller) data, it may be wise to confine these early investigations to more reliable group-based theoretical questions and address individual difference questions as future data becomes available. Here we test the proposition that longitudinal models based on early waves of data can reliably capture different features of developmental trajectories at both the group and individual level.

In this simulation investigation, we generated data and fit models to specifically test this proposition in two primary contexts. First – as a model of the current ABCD data – we tested whether two time point models, as exemplified by a latent change score approach (LCS; Grimm et al., 2012, Kievit et al., 2018), can capture underlying individual trajectory information. Note that this in no way constitutes a critique or test of the latent change score modeling framework itself – readers will note that the LCS can be extended to model full trajectories with ease (Grimm et al., 2012, McArdle, 2009) – it merely allows for the modeling of a two time point difference which is not possible in a traditional latent or multilevel growth curve. Because a two time point model is limited to a fundamentally linear difference score parameterization, we tested performance in the LCS model against known generating parameters in data with a linear functional form over time. In particular, we focused on how reliably these different models capture group- versus individual-difference features of the model. Second — we probed how minimally-defined longitudinal models recovered the generating parameters in both linear and non-linear developmental curves. By minimally defined, we refer to the minimum number of time points needed to identify a curve of a given complexity — 3 for a linear trajectory model, 4 for a quadratic, and so forth (McCormick et al., 2023). With these extended analyses, we hope to inform decisions of future longitudinal research when weighing the number of time points needed to address theoretical questions related to individual differences.

## 2 Approach and results

### 2.1 Simulation approach

For each of our target simulation conditions, we simulated true intercepts and slopes for each individual replicant within a sample, such that each replicant’s true score value is known across an infinite range of observations. We then generated a set of observed scores that combine this true score information with Gaussian noise, resulting in a set of repeated measures for each replicant. For consistency, we scaled the variance of the Gaussian noise to match the observed variance, which has the practical result of maintaining an explained variance of each repeated measure at 50% (equivalent to an $R^{2}=0.50$ in multiple regression).^2^ For each simulation condition, we generated 1000 independent samples to ensure that our effects generalized and did not rely on stochastic features of any one set of sample data. The full details of the simulation can be explored and replicated in the provided Supplemental Code (https://osf.io/9rjcv/).

First, we generated a relatively high *R^2^* value compared to what is commonly found in behavioral or neuroimaging data (Elliott et al., 2020, Funder and Ozer, 2019, Adise et al., 2021, Brieant et al., 2023), increasing the power of the growth model. While significance is not our main concern here, adding excessive noise in the repeated measures (i.e., low *R^2^*) also increases sampling variability of the observed measures within our data, and makes it more difficult to recover reliable individual estimates of trajectories. This can be seen in the sensitivity analyses we conduct in the Supplemental Material (Figures S16-S20), where relatively low $R^{2}$ values for early time points additionally disadvantage 2 and 3 time point models. Additionally, we generated complete case data so that all individual trajectories were estimated similarly and that differential missing data did not impact reliability (see Supplemental Material, Figures S13-S15 for how missing data increase unreliability). Third, we generated data with a known functional form, and correctly specified the fitted model in line with the population-generating model. Of course, matching these two things can be a real challenge in real data – where the true functional form is not known – but here we test the reliability of recovery of fixed effects and individual differences even when we have perfect knowledge of the correct functional form. Finally, we have zero sampling variability at the level of the population-generating parameters across samples. That is, the covariance matrix of the underlying growth trajectory parameters (e.g., intercept and slope) are *identical* across the 1000 samples, and identical to the population values we specify for each condition (detailed below). By reducing this source of sampling variability, we help ensure that our results are not driven by sample characteristics that substantially differ from the population values specified (for instance, samples where intercept and slope are uncorrelated or even negatively correlated). One question we did not explore, however, is whether there is something different about *number* versus *order* of time points. For instance, is using **any 2** time points of data similarly poor at recovering individual parameter values as using the **first 2** time points? However, we feel this is of little relevance to real-world practice, and the main issue is using waves of data before the full longitudinal sample is collected (and thus necessitating the use of the first set of observations).

#### 2.1.1 Model fitting

**Fig. 1:**
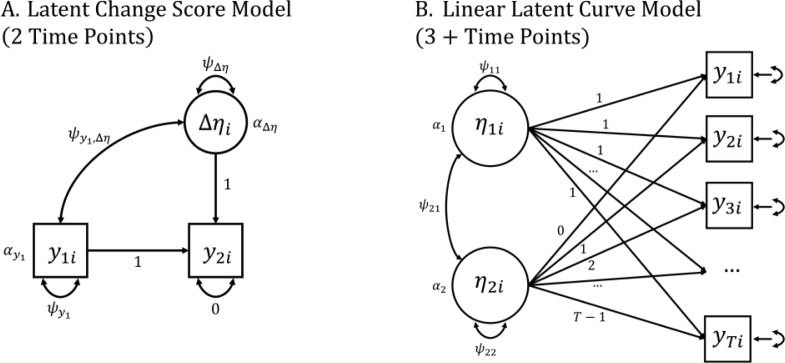
Model Path Diagrams. (A) The two time point latent change score model and (B) the latent curve model were used to fit the data with increasing numbers of waves. Both models include parameters for the means (α’s) and variances (ψ’s) of the initial status ($y_{1i}$ and $\eta_{1i}$) and of change over time ($\Delta \eta_{i}$ and $\eta_{2i}$).

To compare the reliable recovery of individual differences in growth trajectories, we fit a sequence of models to each independent sample data set generated in a given simulation condition using an increasing number of time points available. To facilitate easy comparisons between all the models, we ran all analyses as structural equation models – the latent change score model for the two time point model and latent curve model otherwise – however, the results from the growth models (3+ time points) could equivalently be obtained using a mixed effects modeling approach (McCormick et al., 2023). In each simulation condition, we began with a two time point model, where we fit a univariate latent change score model (LCS; Fig. 1A; e.g., Kievit et al., 2018, Grimm et al., 2012, McCormick et al., 2023). In this model, the latent difference score $\Delta \eta_{i}$ is parameterized by setting both the regression of $y_{2i}$ on $y_{1i}$ as well as the factor loading of $y_{2i}$ on $\Delta \eta_{i}$ to 1. For the 3+ time point models, we fit a standard linear latent curve model (LCM; Fig. 1B; Meredith and Tisak, 1990, McCormick et al., 2023), with an intercept ($\eta_{1i}$) and slope ($\eta_{2i}$) defined by $y_{1i}-y_{Ti}$ (where T is the total number of time points for a given model). The main parameters of interest for capturing trajectories of change are the fixed (group-level mean change; α) and random (individual differences; ψ) effects of $\Delta \eta_{i}$ (i.e., the random difference) and $\eta_{2i}$ (i.e., the random slope) respectively. If the two time point model is a good approximation of the whole trajectory, then the fixed effects should converge on the mean generating value and individual differences in $\Delta \eta_{i}$ should be highly correlated with the individual-parameter values of $\eta_{2i}$ (see McCormick et al., 2023 for a more extensive discussion regarding the contrasts between these two models).

We then examined the change in the distribution across samples of (1) the fixed effect estimate of the slope, and (2) the correlation between individual model-implied slope estimates – or of the change factor in the LCS model – and the true population-generating values. For (2), we also computed the intraclass correlation (ICC(2)) which operates similarly to a correlation, but additionally penalizes the estimate based on differences in the means of the recovered vs. generating parameters. As a simplified example, if the true individual values of the slope are $\eta_{2i}=\left[1,2,\ldots ,50\right]$ and the recovered scores are ${\overset{ˆ}{\eta }}_{2i}=\left[11,12,\ldots ,60\right]$, then their Pearson’s correlation will be perfect (r=1.00) but their intra-class correlation will be penalized (ICC(2) =0.895) because of the mean difference between the two sets of values. As such, the ICC(2) additionally disfavors models that tend to over- or under-estimate the magnitude of effects. Importantly, this approach gives us a within-sample comparison — we can see whether additional observations improve our ability to recover the true individual fixed and individual parameter values within the same data.

### 2.2 Recovery of linear trajectories in typical samples

To begin, we generated data for 200 replicants from a linear latent curve model (Fig. 1B; see Section 1 in Supplemental Code) with 5 repeated measures. This sample size is relatively large for a single-site neuroimaging study (the average ABCD site-specific sample size is 369; Casey et al., 2018; and the median sample size for longitudinal fMRI studies is less than 20; Elliott et al., 2020). As we will see, the exact sample size does not impact the core issues outlined here, so N=200 will give us a sense of what to expect in typical samples. We generated data using the population parameters below.

(1) $$\alpha =\left[\begin{array}{cc} 3 & 0.2 \end{array}\right]\;\Psi =\left[\begin{array}{cc} 1 & \\ 0.15 & 0.25 \end{array}\right]\;\Psi_{std}=\left[\begin{array}{cc} 1 & \\ 0.3 & 1 \end{array}\right]$$

These parameters translate into data where individuals start on average at values of 3 and increase 0.2 units per time point (α contains the factor means), but with significant heterogeneity in individual trajectories for both starting point and rate of change over time (Ψ contains the factor (co)variances; the off-diagonal of the standardized covariance matrix, $\Psi_{std}$, indicates that individual differences in intercept and slope are positively correlated at *r* = 0.3). We then fit the series of models using increasing numbers (2 – 5) of time points.

**Fig. 2:**
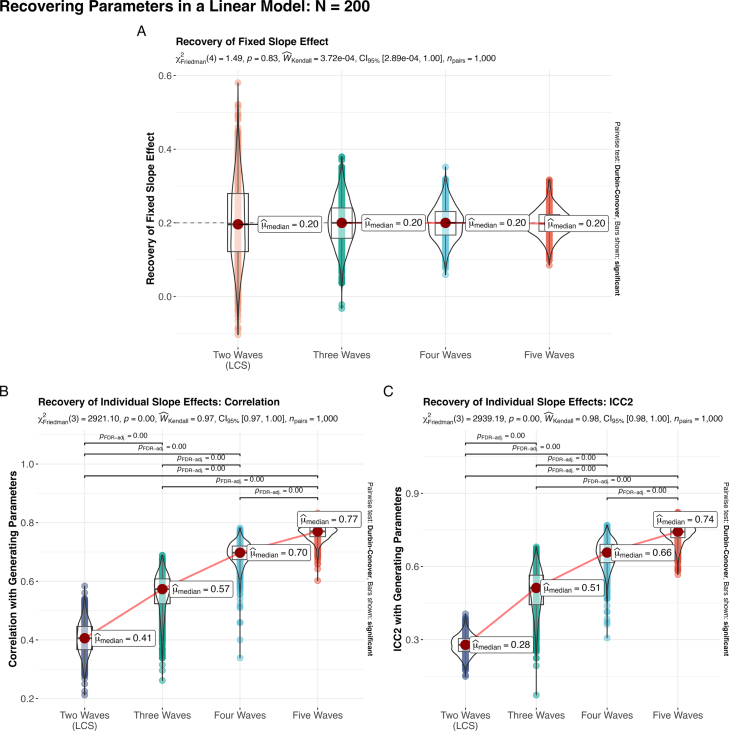
Recovering linear trajectories using increasing waves of data. While the two time point latent change score (LCS) is **(A)** able to recover fixed slope effects on average (dashed line indicates generating parameter value), there are challenges for recovering individual parameter values, with results **(B)** only correlating with the generating parameters at *r*= 0.41 in the median sample (16.8% shared variance). However, even the minimally-defined linear latent curve model on 3 time points does not show reliable recovery of individual differences (*r*= 0.57; *R^2^*= 32.4%). **(C)** There is an even greater deficit for the two time point model in terms of reliability (ICC2), suggesting additional issues with the values recovered in this model.

To begin with the good news, recovery of the fixed effects is relatively reliable, at least on average (Fig. 2A). That is, there is no systematic bias in the recovery of the two time point change score parameter ($\alpha_{\Delta \eta }$) compared with the generating slope parameter ($\alpha_{2}$) from the full growth model, suggesting that under these conditions, we can expect that the two-time point model can serve as a reasonable proxy for the fixed effects we might find in a larger growth model with additional time points. The main caveat with the fixed effects is that the fixed effect recovered in the two time point model (Fig. 2A, far left) has relatively high sample-to-sample variance compared with models which include additional time points (${SD}_{\alpha_{\Delta \eta }}$ = 0.111 vs. ${SD}_{\alpha_{2}}$ = 0.062, 0.045, and 0.035 respectively). This means in practice that results obtained from any given sample will have reduced power (due to an inflated standard error) with a higher likelihood of being biased (in both directions) compared to the median parameter which impacts both type I and type II error rates. However, with appropriate caution, the fixed effects of the two time point model can serve as a useful guide for directing future longitudinal work with additional time points.

In contrast, fitting a two time point LCS model and estimating individual differences in the change score does a relatively poor job of recovering the underlying *individual* slope parameters, with a median correlation between recovered and generating parameters of *r* = 0.41 (Fig. 2B, far left), and even lower median reliability of ICC(2) = 0.28 (Fig. 2C). This model performs poorly despite the change score – necessarily a linear difference – corresponding to the linear functional form of the underlying data, with the recovered scores only sharing 16.8% variance with the generating values. In real sample data, we can likely expect some additional degradation in the quality of individual parameter recovery in cases where the functional form is not strictly linear, data are partially missing, or with noisier measures — unfortunately common conditions in empirical research.

Of course, we should not unfairly demonize the two time point model, because while the 3 time point model does a substantially better job of recovering individual slope parameters, the median sample still only correlates at *r*= 0.57 (*R^2^* = 32.4%; Fig. 2). Instead, only additional time points beyond this specification begins to substantially improve the recovery individual values (Fig. 2). Given the upper bounds due to factor score indeterminacy (Bollen, 2002, Curran et al., 2016 ; most often around *r* = 0.8 – 0.85), we would expect to see some continued marginal gains with additional time points after 5, however, the overall point that additional time points are needed for reliable recovery of individual values seems clear. To obtain reliable estimates of the parameters governing individual trajectories of change, we need to increase the number of waves even beyond minimally-defined models.

The contrast between the correlation and reliability estimates across models is of particular interest here, as it emphasizes a particular limitation of two time point models to recover individual parameter estimates. The ICC(2) measure of reliability not only assesses the preservation of the linear association (like the correlation measure) but also unreliability in the mean of the individual parameter estimates. The additional drop in reliability compared with the correlation suggests the magnitude of the individual effects are not well-captured, in addition to rank order being degraded. The true trajectory models (3+ time points) do not show this same precipitous drop, which seems to be a particular danger of the two time point model.

### 2.3 Recovery of linear trajectories in large cohort samples

**Fig. 3:**
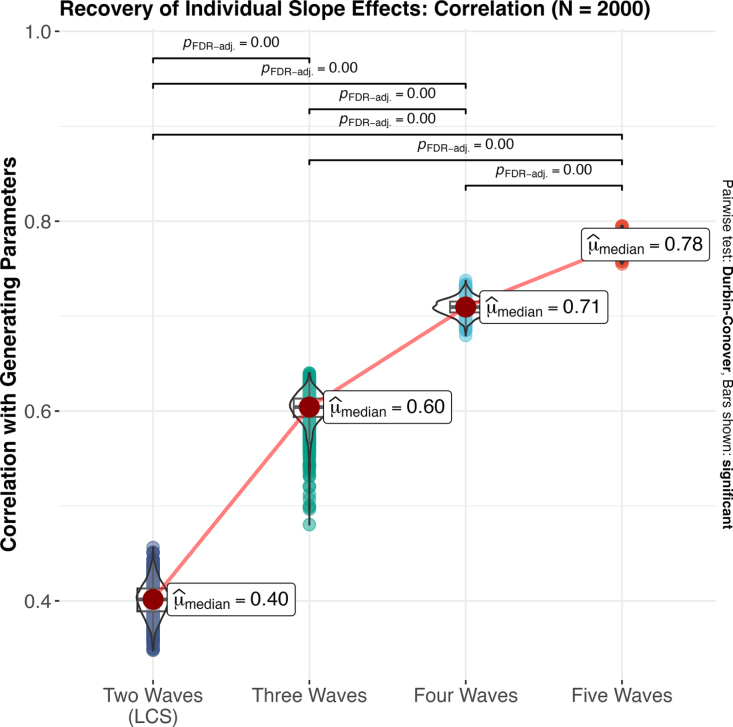
Recovering individual differences in a large cohort using increasing waves of data. While increasing the sample size shrinks the sample-to-sample variability in the recovery of individual differences, it does not impact the median reliability of the results.

Of course, it could be the case that our choice of initial sample size puts the two time point model at a particular disadvantage and that recovery of individual differences would be better in a large cohort design (e.g., Casey et al., 2018, Volkow et al., 2021, Walhovd et al., 2018) that we might expect do a better job of characterizing individual differences. To address this, we increased the size of each of our 1000 simulated samples by an order of magnitude to 2000 individual replicants and then re-ran the same set of analyses (see Section 2 of Supplementary Code). Unfortunately, results suggest that sample size has no appreciable impact on the *median* ability to recover individual slope parameters (Fig. 3; fixed effects continue to be reliably recovered), although the *sample-to-sample variability* is significantly reduced. This same pattern holds for the reliability estimates (Figure S6). As such, we can conclude that while larger samples do reduce the chances that we get especially poor – or especially good – recovered values, relative to the median sample, simply increasing the number of individuals in a study is not sufficient to reliably capture individual differences in longitudinal models. Rather, increasing the number of observations per individual is the key driver of improved recovery.

### 2.4 Recovering individual differences in quadratic model parameters

We can see that in the simple linear change, two time point models can recover fixed (i.e., average or group-level) effects, but do not reliably capture individual differences in change over time, and indeed nor do minimally-defined (i.e., 3 time point) trajectory models do a much better job. These issues naturally extend to more complex trajectories, which we can demonstrate by generating underlying quadratic growth. We used the following population parameters to generate this data (see Section 3 in Supplemental Code).

(2) $$\alpha =\left[\begin{array}{ccc} 3 & 3 & -0.2 \end{array}\right]\;\Psi =\left[\begin{array}{ccc} 1 & & \\ 0.15 & 0.5 & \\ -0.022 & -0.079 & 0.05 \end{array}\right]\Psi_{std}=\left[\begin{array}{ccc} 1 & & \\ 0.3 & 1 & \\ -0.1 & -0.5 & 1 \end{array}\right]$$

This describes data that show initial increases (i.e., instantaneous linear slope) but with negative acceleration over time (i.e., increases tend to level off). In other words, we simulated the “adolescent emergent” trend (Somerville et al., 2013) rather than a full parabola to demonstrate a model where the instantaneous linear slope (i.e., the tangent of the curve where time is coded as 0) was meaningful. This specification offers the possibility that the change score in a two time point model will be related to a slope parameter — that is, the two time point model can at least offer an initial estimate of whether the trajectory is increasing or decreasing over time. Otherwise, the two time point change score will capture different processes at various phases of the quadratic, depending on where in the trajectory the model is estimated. This choice provides the best-case example for a two time point model to meaningfully capture future trajectory results. When we fit the various time point models, we purposely misspecified the 2- and 3-time point models because they cannot support the quadratic component due to insufficient numbers of time points. As such, we only compare quadratic components in the 4 – 6 time point models. However, for the instantaneous linear slope, we can compare how all models might capture that feature of the data.

**Fig. 4:**
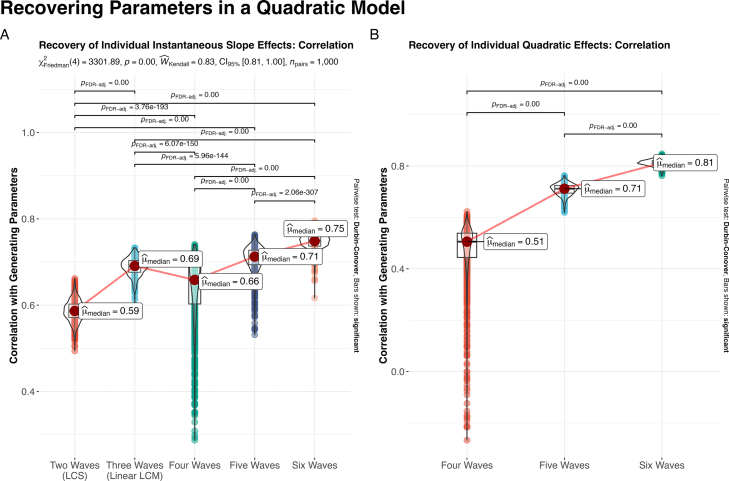
Recovering individual differences in a quadratic model using increasing waves of data. Simulation results show that for both the **(A)** instantaneous linear and **(B)** quadratic curvature parameter, individual differences at the minimally-defined 4-waves are poorly recovered. Instead, over-identified models – with respect to the number of time points – substantially aid reliable recovery.

When we first compare the recovery of this instantaneous linear slope term (Fig. 4A), the reader may notice a pattern that initially seems to conflict with our primary results thus far — that is, there is a small dip in the median recovery of individual difference parameters between the 3- and 4- time point models. The reason for this is that the dimensionality of the 4+ time point models, where we are estimating three growth factors (intercept, linear slope, and quadratic curvature), compared with the 2- and 3-time point models where we only estimate two effects (intercept and slope/latent difference). This increased complexity drives down the reliable recovery of effects, especially in the minimally-defined quadratic model (i.e., 4 Waves, Fig. 4A) — however, note that it still outperforms the two time point model in estimating the linear effect. This transition in model complexity aside, we can see that there are continued improvements with additional time points, and these findings are consistent with the linear model results we have seen already. With the quadratic parameter (Fig. 4B; see Figure S9 for reliability estimates), we see a now-familiar pattern; the model with 5 (${\overset{ˆ}{\mu }}_{median}=0.71$) or 6 (${\overset{ˆ}{\mu }}_{median}=0.81$) waves outperforms the 4 time point model (${\overset{ˆ}{\mu }}_{median}=0.51$) quite substantially (see Fig. 4B for test statistics) in the recovery of individual slope parameters. Additionally, the long tails on the 4 time point model is especially concerning since it indicates that we can get extremely unreliable results in minimally-defined models (the same pattern can be seen for the minimal 3 time point model in Fig. 2). Even the addition of a single extra wave above the minimum required for identification (i.e., 5 time points) has big benefits for reducing the chances of obtaining especially poor recovery.

### 2.5 Modeling more complex growth model relationships

Of course, descriptive unconditional growth models are not the only application of longitudinal data analysis, and many researchers wish to bring in additional covariates (either time-invariant or -varying) and distal outcomes (see McCormick et al., 2023 for a more-expansive discussion of these topics) or specify multivariate models of co- development (Curran and Hancock, 2021). Here the issues outlined thus far are likely to be exacerbated by the more-complex structure of these models and their demands on the data. While the field of developmental cognitive neuroscience relies heavily on mixed-effects models (McCormick et al., 2023), and therefore often uses two-step procedures for relating parameters of growth models with external variables (Liu et al., 2021), within a structural equation model, we can bypass some of the inefficiencies of these procedures and instead directly model the relationship between the parameters of the growth model (Skrondal and Laake, 2001). To demonstrate how this can aid in the reliable recovery of relationships between the parameters of the growth model and an external variable (denoted W for a generic variable), we returned to the conditions in our initial simulation (Section 2.2; N = 200; linear functional form). However, now we simulated an additional variable (W) to correlate with the intercept (r=0.2) and slope (r = −0.1) of the growth trajectory. When recovering parameters, we fit the growth model and correlations with W simultaneously.

**Fig. 5:**
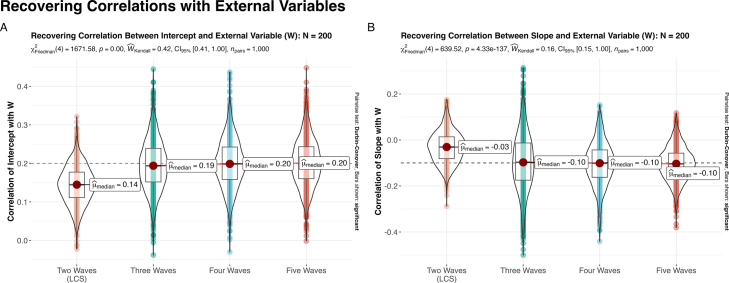
In a model where we simultaneously estimate the growth model and relationships with an external variable W, we can see a specific deficit for the two time point model, where it is biased towards 0 for the relationship with both **(A)** intercept and **(B)** slope. In the 3+ time point models, additional time points aids in reducing sample-to-sample variability but the median effect is unbiased.

When examining the results, we can see that the two time point LCS model shows a specific deficit compared with the trajectory models in capturing the relationship with the external variable (here parameterized as a correlation, but this could be rescaled to a regression weight if desired). That is for the relationship of W with both the intercept (Fig. 5A) and slope (Fig. 5B), the correlation is attenuated towards zero in the two time point model compared with the 3+ time point models. This suggests that even when using simultaneous estimation, two time point models are likely best suited as exploratory analyses — useful for assessing fixed effects, while follow-up analyses with additional waves is likely needed for a reliable understanding of individual differences and relationships with external variables (i.e., covariates and distal outcomes).

## 3 Discussion

Researchers with access to early waves of longitudinal data face a dilemma — interesting and complex research hypotheses can be most reliably answered by waiting for the maximum of time points to be available, but this strategy may be incompatible with pragmatic concerns of grant funding, training goals, and career advancement. As such, it is important to delineate the kinds of research questions that early waves of data can reliably inform, and which require follow-up work when more time points are available. Our results suggest that these early time points of data are more suited to research questions concerning group-level effects. For instance, we can recover the *fixed* (i.e., mean) effect of the growth curve slope reasonably well even in a two time-point model (see Fig. 2A; although caution is still warranted because sample-to-sample variability is high). While not the focus of our simulations here, casual effects like experimental manipulations, interventions, or natural experiments can still offer useful insights in two time point data because they concern the intervening effect of some exogenous factor. Additionally, researchers might have time-constrained hypotheses such as how earlier factors impact the transition to a new context (e.g., pre- vs. post-natal, transition to high school, etc.), where two time points may be able to capture the process of interest. However, for research questions which focus on individual differences in change over time, especially over longer time horizons, additional time points are needed to maximize the reliability of the individual trajectories estimated. We showed evidence that two time point models like the latent change score are unlikely to capture individual differences reliably, sharing relatively little (16.8%) variance with the true generating scores and even lower reliability (ICC(2) = 0.28). The practical implication of this minimal shared variance is the attenuation of relationships that individual trajectory parameters would show with other constructs of interest (Liu, 1988) – indeed we showed that even simultaneous estimation does not ameliorate bias in the estimated relationship with external variables (Fig. 5) – and the low reliability implies that we are not capturing the true magnitude of slope effects. That is, we are minimizing the chance that we can appropriately test our theories of individual differences. Given the high degree of noise in our measures of the brain (Elliott et al., 2020, McEvoy et al., 2000), which will work to attenuate these relationships further, we need additional time points to support the accurate assessment of the course, causes, and consequences of development (McCormick et al., 2023).

### 3.1 How many time points do we need for a given question?

Given the constraints of funding, time, and effort, there is an understandable focus on the *minimum conditions necessary* to fit a model in substantive research. This phenomenon is certainly not unique to longitudinal modeling — the entire practice of power analysis is geared towards finding the minimally-acceptable sample size to detect an effect of interest. However, longitudinal models layer on the additional complexity of the number of time points (Muthén and Muthén, 2002, Schultzberg and Muthén, 2018). These results demonstrate that to recover reliable individual differences, we need to begin moving past this minimum-focused frame and focus analytic strategies on maximizing the number of time points in our longitudinal models. In the interim, we can still utilize emerging datasets for questions that operate at the group rather than individual level, and then follow up on these analyses with individual difference questions once more data becomes available. Rather than viewing this delay as detrimental, however, the field could use this as an opportunity to develop best practices for implementing *cumulative* science, where researchers are encouraged and incentivized to revise, update, and refine prior findings over time — one could almost suggest a developmental approach to the science of development. Importantly, the advantages of additional time points are distinct from increasing the sample size (compare Fig. 2, Fig. 3), suggesting that we need a diversity of data collection strategies (e.g., dense vs. long) to facilitate the full range of developmental questions we wish to address.

We can therefore take it as uncontroversial that more time points are better in theory. But *how much* better, and compared with what? One of the key findings here is how poorly minimally-defined models are in general for recovering reliable individual differences in trajectories. While the two time point model has particular additional disadvantages (16.8% shared variance with the true scores; see Fig. 2, Fig. 5, far left), the three time point model – which is in theory properly specified to recover a linear trend – also struggles to reliably capture individual effects of interest (32.5% shared variance; Fig. 2). In both the linear (Fig. 2) and quadratic simulations (Fig. 4), the use of even one additional time point beyond the minimum required substantially increased the reliable recovery of individual trajectory parameters substantially. As such, we recommend that research with individual differences hypotheses strive to include *at least* one additional time point beyond the minimum requirements for model identification. Additional time points beyond this are certainly even better, since further time points may serve as a buffer against the messiness of real data (especially with missing observations; see Figure S13-S15 in the Supplemental Material).

One final point that we must remain aware of is that while the statistical properties of our model are key for returning reliable inferences about change over time, issues of *design* are even more foundational. That is, we must match the number and timing of measurement occasions to the time course of the phenomenon of interest (Brandmaier et al., 2015, Lerner et al., 2009, Voelkle et al., 2018). For instance, yearly observations can limit our ability to model the course of phenomena such as pubertal development or friendship formation since we would only have very coarse signposts of dynamic processes that occur rapidly. Conversely, however, repeated measurements of ventral striatum reactivity over a week or month are unlikely to capture long-run maturational processes because we do not have the power to detect change at such a short time scale. While some of these decisions are constrained in secondary data analysis, this is nevertheless a foundational question for researchers to ask themselves when planning their analytic plans, and the growing diversity of longitudinal data sets can help by giving researchers multiple data options for addressing their theoretical hypotheses (Kievit et al., 2022).

### 3.2 Potential approaches for ongoing longitudinal neuroimaging data collection

In many existing and emerging data sets (e.g., ABCD, HBCD, YOUth (Onland-Moret et al., 2020), GUTS), researchers are already taking advantage of longitudinal data to address theoretical hypotheses across many domains. We have seen an increase in papers have emerged using the two time points which make longitudinal claims regarding the development of various constructs (we intend to avoid calling out any individual papers or studies in particular, but readers will likely be familiar with examples). With the release of a third wave of data in several of these studies, findings based on longitudinal modeling of these data are likely to dominate many journals in the coming years. Our intention is not to discourage these investigations in their entirety, but to channel these efforts toward questions which can be reliably supported while waiting for additional time points of data to tackle more complex questions of individual differences. As a field, we can also attempt to create new paradigms that acknowledge the need for “slower” science (here limited by the frustratingly slow rate at which youth insist on aging rather than researcher productivity *per se*) while still supporting the advancement of training, career paths, and grant aims. One such strategy might be to create updatable “living” publications as new data becomes available,^3^ or to otherwise incentivize addenda in longitudinal publications after additional waves of data are collected. Similar to proposed sensitivity analyses for including additional time points in growth mixture models to test the robustness in class recovery (Sher et al., 2011), this approach would allow researchers to validate original findings with additional follow-up analyses. Existing tools like registered reports (Hardwicke and Ioannidis, 2018, Klapwijk et al., 2021, Munafò et al., 2017) might also offer a chance for researchers to “stake a claim” on novel research hypotheses (see Barch, 2021 as an example of thinking along these lines), where data analysis occurs after additional time points can be included. We do not pretend to have definitive solutions that address all relevant equities, but a major aim of this work is to stimulate these discussions within fields utilizing longitudinal data — we must begin, as it were, at the beginning.

### 3.3 Conclusions

The fields of psychology, neuroscience, and related fields have undergone many transformations to improve the validity and reliability of findings, from taking seriously measurement reliability, adopting longitudinal data, and increasing sample size. Here we focused on the impact of the number of time points on reliably estimation of effects at the group and individual levels. We showed that two time point data has some particular disadvantages for estimating individual differences, especially when the goal of the analysis is to relate individual growth trajectories to external variables of interest. However, we additionally showed that adopting full trajectory models (3+ time points) addresses some of these issues, there are continued challenges for reliability which further time points beyond the minimum required can work to ameliorate. We propose therefore that early questions using in-progress longitudinal data sets focus on those group-level effects which can guide future individual difference questions as more data becomes available. To support these changes we further propose that institutional and field-wide steps should be taken to encourage practices that maximize the number of time points used in longitudinal research.

## CRediT authorship contribution statement

**Sam Parsons:** Writing – review & editing, Writing – original draft, Visualization, Methodology, Formal analysis, Conceptualization. **Ethan M. McCormick:** Writing – review & editing, Writing – original draft, Visualization, Supervision, Software, Project administration, Methodology, Formal analysis, Conceptualization.

## Declaration of competing interest

The authors declare that they have no known competing financial interests or personal relationships that could have appeared to influence the work reported in this paper.

## Data Availability

All details of the simulation can be explored and replicated in the provided Supplemental Code (osf.io/9rjcv/).

## Footnotes

1 We contrast this to single time point studies where participants are grouped by age in an attempt to investigate age-related differences, which could be described as “cross-sectional stratified by age”, rather than truly longitudinal.

2 We could alternatively set a fixed noise variance across time points, but would need to articulate a rationale for the absolute level chosen. Setting a fixed variance of 1 versus 4 (i.e., a standard deviation of 1 versus 2) impacts the exact parameter correlations or ICCs values, but the pattern of effects remains the same. For figures reflecting these alternative approaches, see the Supplemental Material; however, none of these alternative specifications would change the conclusions we draw here.

3 This may require substantial shifts from the current model of publishing with static PDF output, and so we do not wish to downplay the complexity that some of these changes might require.
